# Rationale, design and methods for a community-based study of clustering and cumulative effects of chronic disease processes and their effects on ageing: the Busselton healthy ageing study

**DOI:** 10.1186/1471-2458-13-936

**Published:** 2013-10-08

**Authors:** Alan James, Michael Hunter, Leon Straker, John Beilby, Romola Bucks, Tim Davis, Robert H Eikelboom, David Hillman, Jennie Hui, Joe Hung, Matthew Knuiman, David A Mackey, Robert U Newton, Lyle J Palmer, AW Bill Musk

**Affiliations:** 1 Department of Pulmonary Physiology and Sleep Medicine/West Australian Sleep Disorders Institute, Sir Charles Gairdner Hospital, Nedlands, Perth, Australia; 2 School of Physiotherapy and Curtin Health Innovation Research Institute, Curtin University, Bentley, Perth, Australia; 3 Pathwest, The University of Western Australia, Crawley, Perth, Australia; 4 School of Psychology, The University of Western Australia, Crawley, Perth, Australia; 5 Fremantle Hospital; School of Medicine and Health, The University of Western Australia, Crawley, Perth, Australia; 6 Ear Science Institute Australia, Ear Sciences Centre; School of Surgery, The University of Western Australia, Crawley, Perth, Australia; 7 School of Anatomy and Surgery, The University of Western Australia, Crawley, Perth, Australia; 8 School of Population Health, The University of Western Australia, Crawley, Perth, Australia; 9 Lions Eye Institute, Centre for Ophthalmology and Visual Science, The University of Western Australia, Crawley, Perth, Australia; 10 Health and Wellness Institute, Edith Cowan University, Joondalup, Perth, Australia; 11 Ontario Institute for Cancer Research and Samuel Lunenfeld Research Institute, University of Toronto, Toronto, Canada; 12 Department of Respiratory Medicine, Sir Charles Gairdner Hospital, Nedlands, Perth, Australia; 13 School of Medicine and Pharmacology, The University of Western Australia, Crawley, Perth, WA, Australia; 14 School of Pathology and Laboratory, The University of Western Australia, Crawley, Perth, WA, Australia

**Keywords:** Busselton, Healthy ageing, Clusters of illness, Cross-sectional, Prevalence, Multi-morbidity

## Abstract

**Background:**

The global trend of increased life expectancy and increased prevalence of chronic and degenerative diseases will impact on health systems. To identify effective intervention and prevention strategies, greater understanding of the risk factors for and cumulative effects of chronic disease processes and their effects on function and quality of life is needed.

The Busselton Healthy Ageing Study aims to enhance understanding of ageing by relating the clustering and interactions of common chronic conditions in adults to function. Longitudinal (3–5 yearly) follow-up is planned.

**Methods/design:**

Phase I (recruitment) is a cross-sectional community-based prospective cohort study involving up to 4,000 'Baby Boomers’ (born from 1946 to 1964) living in the Busselton Shire, Western Australia. The study protocol involves a detailed, self-administered health and risk factor questionnaire and a range of physical assessments including body composition and bone density measurements, cardiovascular profiling (blood pressure, ECG and brachial pulse wave velocity), retinal photography, tonometry, auto-refraction, spirometry and bronchodilator responsiveness, skin allergy prick tests, sleep apnoea screening, tympanometry and audiometry, grip strength, mobility, balance and leg extensor strength. Cognitive function and reserve, semantic memory, and pre-morbid intelligence are assessed. Participants provide a fasting blood sample for assessment of lipids, blood glucose, C-reactive protein and renal and liver function, and RNA, DNA and serum are stored. Clinically relevant results are provided to all participants. The prevalence of risk factors, symptoms and diagnosed illness will be calculated and the burden of illness will be estimated based on the observed relationships and clustering of symptoms and illness within individuals. Risk factors for combinations of illness will be compared with those for single illnesses and the relation of combinations of illness and symptoms to cognitive and physical function will be estimated.

**Discussion:**

This study will enable a thorough characterization of multiple disease processes and their risk factors within a community-based sample of individuals to determine their singular, interactive and cumulative effects on ageing. The project will provide novel cross-sectional data and establish a cohort that will be used for longitudinal analyses of the genetic, lifestyle and environmental factors that determine whether an individual ages well or with impairment.

## Background

Substantial rises in the prevalence of many chronic diseases and degenerative conditions such as cardiovascular disease, cancer, diabetes, depression, dementia, asthma, chronic obstructive pulmonary disease (COPD), arthritis, osteoporosis, sarcopaenia, hearing loss and vision disorders have occurred in Australia and other developed countries over the last 3 decades [1]. In 2003, approximately 20% of Australians (~4 million people) reported having a chronic condition leading to disability [1] and this percentage is likely to rise substantially if current trends of increased rates of obesity and decreasing physical activity in the general population continue. In Australia, like many other nations, the mean age of the population has been increasing. By 2050, it is estimated that almost one quarter of the population will be aged 65 years or older [2].

Australian surveys conducted in 2004 indicate that approximately 30% of adults aged between 45 and 64 years had 5 or more long-term co-morbidities, a percentage that increased to 55% for those aged 65–85 years, and to 65% for those over 85 years [3]. Concurrent chronic and degenerative diseases are associated with significant long-term morbidity and mortality, and are a major public health challenge for Australia and the rest of the world [4]. For example, the health burden from back pain with depression is substantially greater than back pain without depression [5]. An ageing population that has a disproportionate number of individuals with one or more co-morbidities will place significant demands on the health system, the first effects of which will likely be seen in the near future as the current baby-boomer generation (born 1946–1964) reaches ages associated with the highest rates of morbidity. In the context of health systems that are already under pressure, there is an urgent need to assess the global effects of concurrent illnesses to identify the most effective strategies for intervention and prevention.

In recent years, there have been efforts to define what constitutes 'healthy ageing’ [6]. Studies have assessed measurable constructs that contribute to overall quality of life and include the absence of disability and chronic disease, the maintenance of optimal physical and cognitive function and the importance of psychological health and social connectedness. The search to define a 'healthy ageing’ phenotype [6] has placed an emphasis on an integrated multidisciplinary approach with recommendations that research efforts focus on a number of areas including: physical activity, fitness and sleep, effects of well-being or psychological health on biological mechanisms of disease and resilience, prevention strategies through combined lifestyle and pharmacological interventions, and defining reversible changes prior to disease and disability through the identification of new biomarkers [6]. Underpinning these recommendations, is a growing recognition that a number of age-related disease processes are likely to arise from common pathways and perhaps provide opportunities to maximise the benefits of intervention and prevention. For instance, age-related hormonal changes or lifestyle factors such as obesity, malnutrition and lack of physical activity may exacerbate inflammation and cellular damage leading to metabolic dysregulation. Metabolic dysfunction is a common process that may underlie a range of conditions and the progression to chronic disease. Examples include the progression of insulin resistance to diabetes, atherogenesis and dyslipidemia to cardiovascular disease, neurocognitive decline to dementia, and osteopenia to osteoporosis [6].

By understanding the trajectories of chronic diseases and the nature of the complex relationships amongst them, insights can be gained into the impact of combinations of illnesses and symptoms on function and quality of life. This will inform the development of strategies that focus on the maintenance of function to better ensure engaged, productive and healthy ageing of individuals. It is increasingly clear that chronic disease research and, by extension, management of the burden and risk of future chronic disease, must be approached holistically rather than on a disease-by-disease basis.

There have been a number of recent cohort studies which have examined the burden of disease in ageing populations. Some of these have necessarily focused on specific diseases [7-9] and some have been confined to a particular sex or to older subjects [10,11] or have been limited (due to very large samples) to questionnaire-derived data [12]. The Busselton Healthy Ageing Study (BHAS) will use a multi-disciplinary approach to understand the impact of illness on healthy ageing. The strategy is to study a moderately-large cohort intensively, by collecting a wide range of measures of physical function, cognition, well-being, quality of life and medical history as well as biological samples in order to discover the important relationships between disease processes and symptoms within individuals. The study will establish a cohort for investigations into the development of morbidity and reduced function and quality of life into old age. The generation born from 1946 to 1964 (baby boomers) offers an ideal sample to study the factors associated with ageing. The 20-year interval between the youngest and oldest members in this cohort provides an opportunity to investigate both early and established disease processes and trajectories associated with ageing and to identify early biomarkers of chronic or degenerative conditions. The BHAS will also establish a platform from which early intervention strategies can be implemented and evaluated. Phase 1 of the study is designed around the following hypotheses, that:

1. multiple physical and cognitive illnesses and their risk factors contribute to the total burden of disease and that there is considerable variation in the burden of disease (within age- and sex-matched individuals);

2. the overall burden of illness in an individual is determined not just by specific illnesses but also by the combination of illnesses and their aggregate effects;

3. quality of life and physical and cognitive function are partly determined by various factors (socio-demographic factors and illness risk factors) that contribute to the total burden of illness and;

4. the burden of illness, together with measures of physical and cognitive function and quality of life, are associated with health care utilization, employment and occupation, residential setting and community participation.

### Study design

The Shire of Busselton is a coastal community in the South-West of Western Australia with a relatively stable population of predominantly European descent. The Shire has been the site of repeated cross-sectional surveys of adults from 1966 to 2009, with participation rates ranging from 64 to 91% [13]. Health surveys have previously focused on specific systems or diseases: respiratory [14,15]; cardiovascular [16-18]; gastrointestinal [19]; endocrine [20]; iron metabolism [21,22]; cancer mortality [23]; the “metabolic syndrome” [24]; dementia and diabetes [25] and genetic epidemiology [13,26-28].

Phase I of the BHAS is a cross-sectional study of community-dwelling adults, born 1946 to 1964 and randomly selected from the Shire electoral roll. Longitudinal follow-up of the same cohort is planned every 3–5 years (Phase II). The study has received ethics approval from the University of Western Australia Human Research Ethics Committee (Number RA/4/1/2203) and participant recruitment commenced in May 2010.

### Aims

The general aim of the BHAS is to identify the cumulative effects of disparate illnesses that constitute the burden of disease that impacts on healthy ageing, starting with a detailed assessment of physical function, cognitive performance and quality of life in baby boomers’.

The specific aims of Phase 1 of the study are to:

1. Determine:

a) Prevalence of chronic physical and mental illness

a) Prevalence of potential multiple-domain risk factors

a) Quality of life and health burden, in 4000 baby boomers from a community-dwelling population sample.

2. Determine the clustering within individuals of:

a) physical and mental illnesses

a) potential risk factors.

3. Determine clustering, across individuals, of:

a) physical and mental illnesses

a) risk factors

a) burden of illness, based on age, sex, and socio-economic factors.

4. Examine associations between illness clusters and risk factor clusters.

5. Collect and store serum, DNA and RNA from blood samples for future studies of genetic risk factors and gene-environment interactions and for the identification of biomarkers associated with ageing and disease.

6. Provide a baseline for follow-up assessments to enable analysis of changes in risk factors and outcomes with a view to determination of factors that impact on healthy ageing, healthcare utilization, employment and occupation, residential setting, and community participation.

## Methods/design

### Study sample and recruitment process

The Busselton Shire comprises about 1.25% of the Western Australian population (25,355 people according to the 2006 Australian Census) and a total of 6,690 adults born from 1946 to 1964 are registered on the 2010 electoral roll in the region, with 48.4% being male and an average of 352 individuals per year of birth group. All non-institutionalised baby boomers who currently live in the Shire and listed on the electoral roll are eligible to participate. Order of invitation to participate is randomized, with recruitment efforts focused upon sequential 10% sample draws. Contact with participants is via a letter of introduction, followed by a phone call to invite them to attend the testing centre for a four hour appointment. Addresses are obtained from the electoral roll and phone numbers are obtained from local residential and business directories.

Phase I of the BHAS commenced mid-2010 and will run until 2013. Based on current recruitment progress and participation rates, it is anticipated that approximately 4,000 participants will be recruited and tested (59% of those listed on the electoral roll).

### Questionnaire data collection

All participants who attend the testing centre complete a self-administered questionnaire that collects information on the following:

#### Demographics

Age, sex, marital status, ancestry/ethnicity, highest education level obtained and age at completion, current and previous occupational history (including years spent in longest occupation and exposure to dust), household income, dwelling type, years lived at current dwelling and occupancy status.

#### Lifestyle, activity and environment

Information on smoking history and exposure to environmental tobacco smoke at home or work is recorded along with alcohol consumption, dietary intake of meat, fruit, vegetable, dairy and cereals and also foods avoided or never eaten.

Physical activity and sedentary behaviour is assessed using the short form of the International Physical Activity Questionnaire*, IPAQ*[29]. The average number of hours of sleep obtained over a typical 24 hour period, exposure to occupational noise, and information on household floor coverings and pet ownership is also collected.

Information technology exposure including mobile phone, television, computer and internet usage is assessed using the Young People’s Activity Questionnaire [30]. Attitudes to information technology are assessed with the computer confidence subscale of the Computer Attitudes Scale [31] that has independently verified factor constructs [32].

Information on social inclusion and community values is collected using nine items from the Social Capital Questionnaire (SCQ) [33], which encompasses constructs of “*feelings of trust and safety*”, “*neighbourhood connections*” and “*proactivity in the social context*”. Information on life values, including self-reported importance of financial security, social and job status and personal relationships is also collected.

#### Medical history

Current use of prescribed and non-prescribed medication and supplements are collected along with medical history including age at doctor-diagnosis of the following diseases and conditions:

•*Cardiovascular conditions* including: angina, claudication, high blood pressure, high cholesterol, implant of cardiac pacemaker, heart attack, transient ischaemic attack, stroke,

•*Endocrine conditions* including: diabetes (Type I, II or gestational), osteoporosis, kidney disease and thyroid disease.

•*Neurological conditions* including: Alzheimer’s disease, vascular dementia (multi-infarct dementia), Parkinson’s disease, attention deficit (hyperactivity) disorder, anxiety disorder including post traumatic stress disorder, depression, bipolar disorder, schizophrenia and epilepsy.

•*Allergies and respiratory conditions* including: asthma or bronchial asthma, chronic obstructive pulmonary disease, pneumonia, pleurisy, bronchitis, sinusitis, hay fever or allergic rhinitis, food allergies and anaphylaxis.

•*Sleep disorders* including: narcolepsy and obstructive sleep apnoea.

•*Gastrointestinal disorders* including: stomach (gastric) or duodenal ulcer, colon cancer, colonic polyps, coeliac disease, gastro-oesophageal reflux disease, hiatus hernia, Crohn’s disease, ulcerative colitis (or proctitis), irritable bowel syndrome, diverticular disease, gallstones and haemorrhoids.

•*Other medical conditions* including: cancer, chronic ear infection, Ménière’s disease, macular degeneration, glaucoma, cataract, injury or trauma resulting in loss of vision, trauma to the head or neck, migraine, headache, anaemia, arthritis, cirrhosis of the liver, fatty liver, poliomyelitis and urinary tract infection.

Information on the age of each participant’s biological parents and siblings and cause of death (if deceased) is collected along with family history of common medical conditions including asthma, diabetes, hay fever, hearing loss, high blood pressure, myocardial infarction, stroke, glaucoma, macular degeneration and cancer. Consent is also requested from participants to allow their coded study information to be linked to information routinely collected by the Western Australian Department of Health, such as hospital admissions, and other government departments responsible for births, deaths and marriage records.

#### Physical symptoms

The questionnaire also collects information on a range of current symptoms including the following:

•*Spinal pain:* Location, frequency, duration and intensity of back pain is assessed based on the Nordic Questionnaire which has demonstrated reliability and validity [34], with modifications based on subsequent research [35-37]. The impact of low back pain on daily activity and physical activity as well as health care use, medications taken, health professionals visited and work/study days lost [38]. Beliefs about spinal pain are assessed with the Fear Avoidance Beliefs Questionnaire [39], the catastrophising subscale of the Coping Strategies Questionnaire [40] and the Back Pain Beliefs questionnaire [41]. Disability related to low back pain is assessed with the Oswestry Disability Index [42] and items from the Örebro Musculoskeletal Pain Questionnaire [43].

•*Respiratory symptoms:* Incidence and frequency of wheeze, breathlessness (at rest or on exertion), chest tightness and cough and phlegm are collected using standardized questions from the British Medical Council Respiratory questionnaire [44].

•*Sleep symptoms:* Information on day-time somnolence, snoring frequency, witnessed apnoeas, frequency of unrefreshed sleep and waking tired or falling asleep while driving is assessed using the Epworth Sleepiness Scale [45] and the Berlin Questionnaire [46].

•*Hearing impairment:* Frequency and effect of hearing loss/impairment, tinnitus, hyperacusis (intolerance to sound), imbalance and dizziness on daily life and activities are collected.

•*Mental health*: Current level of depression, anxiety and stress are assessed using the Depression, Anxiety and Stress Scale (DASS21) [47] and depressive severity is measured using the Patient Health Questionnaire - 9 (PHQ-9) [48]. Information on current treatment for depression including medication, exercise or psychological counselling is also collected.

#### Quality of life

Self-reported health rating, long standing disability or illness and the impact of physical and mental function on daily activities is assessed using the Short Form SF-12® Health Survey [49].

### Study centre data collection

Participants spend up to four hours at the study centre in Busselton where they provide a fasting blood sample and undergo comprehensive physical and cognitive assessments. The order of tests is specifically scheduled to minimise possible confounding interactions (Table 1).

**Table 1:** Schedule of testing in the Busselton healthy ageing study

	**Test**	**Components**
1	Specimen collection	Fasting blood sample and oropharynx swab
2	Anthropometry	Height*, weight*, neck, hips, waist, Mallampati score and pharyngeal grade
3	Participant break	Refreshment break with food provided
4	Cognition	MMSE, AusNART, category and letter fluency test, CDR battery
5	Body composition	Anterior- posterior spine, dual femur and total body densitometry scans*
6	Eyes & vision	Tonometry, keratometry, autorefraction, retinal photography
7	Cardiovascular	12 Lead ECG*, vascular profiling (blood pressures, pulse wave velocity)
8	Ears & hearing	Otoscopy, tympanometry, audiometry*
9	Respiratory	Baseline spirometry*
10	Allergy	Forearm skin prick test*
11	Physical tests	Force plate jump, balance, timed up and go, chair rises, grip strength
12	Bronchodilator response	Spirometry >15 minutes post salbutamol*
13	Sleep monitor instruction	Sleep monitor instruction and overnight sleep study performed in-home*

*Results provided to participant.

*Abbreviations*: *AusNART* Australian National Adult Reading Test; *CDR* Cognitive Drug Research; *ECG* Electrocardiogram; *MMSE* Mini Mental State Examination.

#### Blood sample

A fasting venous blood sample is collected from the cubital fossa in evacuated clot, fluoride/oxalate, ethylenediaminetetraacetic acid (EDTA) and gel serum separator tubes (SST) (BD Biosciences, Australia). Whole blood is also collected in PAXgene® blood tubes for preservation of RNA for later gene expression studies. A range of haematological and biochemistry tests (Table 2) is conducted on fresh samples by PathWest laboratories (Busselton and QEII Medical Centre, Nedlands, Western Australia). Serum, plasma and buffy coat fractions are processed and separated onsite for biospecimen banking. DNA and RNA will be extracted and stored by the Western Australian DNA Bank.

**Table 2:** **Haematology**, **biochemistry and biological specimen banking in the Busselton healthy ageing study**

Haematology*	**Analysate**
Red blood cells	Haemoglobin
	Red corpuscle count
	Haematocrit
	Mean corpuscular volume
	Mean corpuscular haemoglobin
	Mean corpuscular haemoglobin concentration
	Red blood cell distribution width
White cell count	Total count
	Differential count
Platelets	Count
	Mean platelet volume
Biochemistry*	
Urea & electrolytes (plasma)	Sodium
	Potassium
	Bicarbonate
	Urea
	Creatinine
	Estimated glomerular filtration rate
Liver function tests (plasma)	Total Protein
	Albumin
	Globulins
	Bilirubin
	Alanine transaminase
	Alkaline phosphatise
	Gamma glutamyl transferase
Lipids (plasma)	Cholesterol
	Triglyceride
	Low density lipoprotein-cholesterol
	High density lipoprotein (HDL)-cholesterol
	Cholesterol/HDL ratio
General chemistry (plasma)	C-reactive protein
	Glucose
	Insulin
General chemistry (whole blood)	Glycated haemoglobin
**Bio-specimen banking#**	
Buffy coats	DNA extraction and analyses
PAXgene® Blood tubes	RNA extraction and expression analyses
Oropharynx swabs	Bacterial DNA extraction and analyses
Serum	Aliquots for biochemistry
Plasma	Aliquots for biochemistry

*Results provided to participant; # Specimens stored at -80°C.

#### Anthropometry and body composition

Standing height, neck, waist and hip girth, and weight are measured with the participant lightly-clothed and shoeless using standard anthropometric techniques. Upper airway (oropharaynx) anatomy is visually examined and Mallampati and pharyngeal grading is scored [50]. Body mass index (BMI) is calculated as well as waist to hip ratio (WHR). Dual energy X-ray absorptiometry (DEXA) scans (AP spine and dual femur) are undertaken to assess bone mineral density (BMD) using a GE Lunar Prodigy Pro densitometer and enCORE Version 13 (GE Health) software. Bone mineral density is measured in grams per centimetre squared (g/cm^2^) and young adult T-scores and age-matched z-scores are derived using the combined Geelong/Lunar reference database (GE Health). Full body densitometry scans are performed to measure regional body fat distribution, total lean tissue, whole body bone mineral content (BMC) and fat mass. The percentage of total body fat, android fat, gynoid fat, and the android/gynoid ratio are calculated. All body composition scans collected can be manipulated to define custom regions of interest for specific fat compartmentalisation or distribution analyses.

#### Cardiovascular

A resting 12-lead electrocardiogram (ECG) and rhythm strip is recorded digitally using a CardioPerfect PC-based resting ECG (Welch Allyn). Blood pressure in all four limbs is measured with the participant supine, after 5 minutes of rest. Pulse, systolic, diastolic and mean arterial blood pressures are measured using a vascular profiler (Omron VP1000). Estimations of arterial occlusion and distensibility are derived from the ankle brachial index and brachial-ankle pulse wave velocity measurements. Vascular profiling is not performed on participants with recent thrombosis or lymphadenectomy or other conditions in which application and inflation of the cuffs is likely to cause discomfort or exacerbations.

#### Respiratory and allergy

Forced expired volume in one second (FEV_1_) and forced vital capacity (FVC), before and 15 minutes after salbutamol (200 mcg) delivered via a metered-dose inhaler and spacer, are measured using an Easyone^TM^ spirometer and compared with predicted values [51]. Participants are asked to withhold short-acting inhaled bronchodilators for at least eight hours prior to spirometry and long-acting bronchodilators for 48 hours where possible. Spirometry is rescheduled if the participant has undergone eye surgery or has had a detached retina, heart problems or abdominal or chest surgery in the last 3 months, or has had influenza or a chest infection within the last 2 weeks. Swabs of the posterior wall of the oropharynx are collected and stored for later bacterial identification by DNA sequencing. DNA will be extracted from the swabs and submitted to PCR amplification of the V3-V5 variable region of bacterial 16S rRNA. Skin prick tests to common aero-allergens (Grass Mix #7, Rye Grass Pollen, dust mites: *Dermatophagoides pteronyssinus* and *Dermatophagoides farinae*, Cat-Pelt, Dog Hair Dander, Mould Mix #10, *Alternaria tenius* and *Aspergillus fumigatus* (Stallergenes, France)) is performed on the anterior forearm. Wheals 3 mm, or greater, in diameter measured at 15 minutes post-application and pricking are scored as positive reactions. Negative (glycerine) and positive (histamine) controls are used. Participants are asked to refrain from taking antihistamine medications for at least 1 week prior to allergy testing, where possible.

#### Ears and hearing

Otoscopic examination of the external auditory canal is performed using a Mini 3000 otoscope (Heine, Germany) and visibility of the tympanic membrane, outer ear structures, obstructions and discharges are noted. Ear canal volume, middle ear pressure and compliance of the tympanic membrane are measured using an Otowave tympanometer (Amplivox Ltd, United Kingdom). Tympanometry is rescheduled if a participant has undergone ear surgery, perilymph fistula or barotrauma within the previous three months. Pure tone air and bone conduction thresholds are determined using automated audiometry with AMTAS software (AMTAS, Audiometry Incorporated, USA) controlling a Conera audiometer (GNOtometrics, Copenhagen, Denmark), with Sennheiser HDA 200 (Wennesbostel, Germany) headphones, on a NOAH platform (Hearing Instrument Manufacturers’ Software Association, Copenhagen, Denmark) and conducted in a sound-proof booth (Acoustic Designs, Australia) [52]. The participants are first instructed through the headphones. The testing protocol is in accordance with Hughson-Westlake methods. Participants are asked to respond with “Yes” or “No” after the presentation of every stimulus using a touch screen. Air conduction thresholds are recorded at 250, 500, 1000, 2000, 4000 and 8000 Hz, and bone conduction thresholds at 500, 1000, 2000, and 4000 Hz.

#### Eyes and vision

Intraocular pressure (IOP) is measured using a rebound tonometer (Icare, USA) and the average of 6 measurements for each eye is recorded in millimetres of mercury (mmHg). Keratometric and autorefraction measurements are taken using a handheld Auto Ref/Keratometer ARK-30 (Nidek, Japan) to assess refractive errors and to determine corneal curvature radius (corneal refractive power). A high-resolution digital retinal camera (Canon CR-1, Japan) is used to capture two images, one centred on the optic disc and the other centred on the macula. These images are assessed for pathological features of age-related macular degeneration and glaucoma. All eye assessments are conducted after dilation of the pupil using a mydriatic solution (tropacamide 1%) administered 15 to 20 minutes prior to testing.

#### Physical function, neuromuscular strength and power

Dynamic muscle power and strength of the leg extensors are assessed using a Ballistic Measurement System (Fitness Technology, Australia) which incorporates a force plate (400 series, Fitness Technology, Australia) for measurement of ground reaction force [53]. Participants are asked to perform a countermovement vertical jump for maximum height from the force plate and the highest power output and force reproducible to within 5% from up to 8 trials is recorded. A number of parameters are measured or derived including peak and average force, impulse, peak and average power output, peak velocity, maximum rate of force development, and jump height. Participants with current back, hip, knee or ankle pain may withdraw from the jump test at their discretion. Mobility is measured using the timed up and go (TUG) test [54]. Lower-body muscle power and balance is also assessed using the Five Times Sit to Stand Test (5TSTS) [55]. The test involves recording the time taken for the participant to perform 5 successive chair rises from a seated position with arms folded across the chest. Hand grip-strength is measured in kilograms using a hand dynamometer (Model 78010, Lafayette Instrument, USA) with the results of 3 trials (within 5%) recorded for each hand.

#### Balance and risk of falling

Static balance is assessed using a force plate (400 Series, Fitness Technology, Australia) with vertical forces analysed to determine the centre of pressure on the plate. A modified clinical test of sensory interaction on balance involves the participant standing stationary for 30 seconds on the force plate during four conditions; a) eyes open and b) eyes closed, while standing on a stable surface, *and* c) eyes open and d) eyes closed, while standing on an unstable surface [56]. During these trials movement of the centre of pressure and postural sway is recorded using Inner Balance software (Innervations, Australia).

#### Cognition

Cognitive testing focuses on episodic memory assessment, as well as tests of attention and executive function. Memory and attention is assessed using the Cognitive Drug Research (CDR) [57] computerised assessment system (United BioSource Corporation, UK). This has been used extensively in clinical and longitudinal studies, including the dementias, over the last 20 years, has been shown to be sensitive to subtle cognitive changes, and has the largest normative database of its kind [57]. The battery consists of several key tasks including: immediate and delayed word recall and recognition, simple and choice reaction time, digit vigilance, spatial and numeric working memory and delayed picture recognition. Participants also complete measures of retrieval ability and executive functioning using the phonemic (letters C, F, L) and semantic (category - animal) fluency test with trials conducted over 1 minute [58]*.* Pre-morbid intelligence is estimated from scores on the Australian National Adult Reading Test (AusNART) [59] and used as an index of cognitive reserve. Participants also complete the Mini Mental State Examination (MMSE) [60].

#### Sleep-disordered breathing

Sleep apnoea screening devices are offered to participants to use over one night at home. Sleep studies are undertaken using dual channel (oximetry & nasal pressure) ApneaLink™ devices (Resmed, Australia). Participants are instructed on how to fit and operate the units and are also provided with an instruction card and tape for cannula attachment to the cheek. The monitors are returned to the study centre the following day. Data are downloaded and automatically analysed using ApneaLink software version 8.00. Scores of respiratory disturbances including snoring events, apnoea hypopnea index and oxygen desaturation and duration are recorded. Each participant is provided with a score sheet and explanatory guide to indicate the level of sleep–disordered breathing based upon risk index scores at different cut-offs (normal <5, mild 6–20, moderate 21–30 and severe >30). Participants who receive current CPAP or mandibular advancement splint therapy for sleep apnoea are excluded from the sleep apnoea screening study.

### Information provided to participants

Following completion of the testing protocol and return of the questionnaire, participants receive an explanatory guide and copy of the following test results: height, weight, waist measurement, haematology and biochemistry, lung function, skin test responses, cardiovascular profile (blood pressure and ankle-brachial index), bone density, audiometry and sleep apnoea screening test. All results from the physical tests are discussed with the senior scientist onsite and blood tests are posted to participants after being reviewed by the study chief clinical investigator. Participants are urged to provide a copy of their results to their general practitioner where indicated.

### Data capture and management

Hard copies of questionnaires are converted to electronic portable document file (PDF) format and data are extracted and verified on-site using Cardiff TELEform software (Verity Inc. Sunnyvale, CA). These data, along with automatic data capture capabilities from most of the devices being used are stored with the existing Busselton Health Study collection which is managed by the School of Population Health, University of Western Australia.

### Quality control

Each study participant is taken through the testing protocol by a single research assistant. Therefore, all research assistants are trained in all aspects of testing. Training is conducted on-site and, where necessary, within the laboratories of each of the participating investigators under the supervision of experienced laboratory staff, until the required standard of testing and competency is achieved. Raw data are reviewed weekly in-house or within the laboratories of investigators to ensure that standards are maintained, with regular feedback to research assistants and further instruction or adjustments provided where necessary. Complex interpretation and review of results from retinal photographs and bone density scans are undertaken off-site by experienced, specialised staff within the participating laboratories. All equipment is maintained, serviced and calibrated according to manufacturer’s specifications and schedules.

### Data analysis and statistical power

It is expected that most analyses will be based on adequate data for about 4,000 people (with about equal numbers of men and women and with relatively uniform numbers across the year-of-birth groups). The statistical approaches used to investigate the aims of the BHAS Phase 1 are:

#### Estimation of the prevalence of multiple chronic disease processes

Questionnaire results and the results of physical and cognitive testing will be recorded to calculate the prevalence of diagnosed illness, medication use, current symptoms, current cognitive and physical functional status, quality of life and risk factors for disease. Logistic regression and linear regression methods will be used to compare and analyse trends in prevalence and mean levels and to investigate risk factors for illnesses. With 4,000 participants, there will be an average of 105 people in each sex-year-of-birth group and this will allow the prevalence (as a proportion) in each group to be estimated with standard error of 0.03 to 0.04 (for prevalence proportions ranging from 0.1 to 0.25) and much more accurately when groups are aggregated or estimates are based on fitted logistic models. This sample size will provide over 85% power to detect differences as small as 0.05 in prevalence proportions (ranging from 0.1 to 0.25) between men and women or between groups defined by risk factors and symptoms with prevalence around 25%.

#### Determination of the aggregation, clustering and distribution of illnesses (burden of illness)

Burden of illness will be based on the number and specific types and combinations of illnesses. Frequency (contingency) tables and logistic regression methods will be used to investigate relationships between illnesses and how these vary across socio-demographic groups. Generalised linear regression methods will be used to identify risk factors for number and combinations of illnesses (and symptoms) and compare these with the standard risk factors for individual illnesses. Cluster analysis methods (e.g. k-means cluster and latent class analysis) will be used to identify clusters of people who are homogeneous with respect to risk factors and symptoms and then contingency table methods will be used to explore the relationships between the clusters and specific illnesses and combinations of illnesses.

#### Physical and cognitive function and quality of life and relationship to the burden of illness and risk factors for illnesses

Generalised linear regression models using the SF-12® Health Survey quality-of-life score and measures of cognitive function (memory, attention, executive function, category and letter fluency and reading test) and physical function (static balance, chair-rise, timed up and go, muscle strength and power) as outcomes will be used to examine the relationship of illnesses (and burden of illness), symptoms and risk factor variables with these outcomes.

#### Future longitudinal analyses

Changes from baseline to four-year follow-up in risk factors, socio-demographic factors and living arrangements, and outcomes (physical function, cognitive function and quality of life) will be measured and relationships between them investigated using standard longitudinal data regression methods (such a generalised mixed models and GEE estimation). The focus of these analyses will be to understand the patterns and processes of healthy (and unhealthy) ageing and the identification of factors that are associated with healthy ageing.

## Discussion

The impact and costs of failing health are predicted to be a significant burden on health systems and society over the coming years [61]. For example, preventable, non-communicable chronic diseases are now responsible for over 70% of the illness and injury experienced by the Australian population, projected to rise to 80% by 2020. Internationally, all levels of Government are searching for ways to manage this financial and social imperative. Preventive medicine strategies, management of illness, health planning and optimal use of the health system rely on relevant, current and integrated data on the prevalence and clustering of illness, the prevalence and clustering of known risk factors, the discovery of new risk factors and the effect of these diseases and risk factors on function and quality of life. The BHAS will add new knowledge across a broad range of areas by:

1. *Measuring current illness and risk factors in an older general population sample.* This will provide contemporary prevalence estimates of common diseases and symptoms in a community-dwelling cohort as well as provide information on the prevalence of under-researched conditions that affect this age-group including hearing loss, spinal pain, depression and cognitive impairment. The cross-sectional and random recruitment design, coupled with good response rates will maximize the applicability of the results to the general baby-boomer population.

2. *Analysing the cumulative impacts of clustering of illness and risk factors on function*. In order to improve the early stage diagnosis, treatment, and prevention of chronic diseases associated with ageing, it is imperative to gain an understanding of the complex relationships among common diseases. The wide range of both objective and subjective measures collected will allow the identification of novel associations and clustering of diseases within individuals and determine their relative importance in reduced function and quality of life.

3. *Measuring a number of biomarkers and storing serum, DNA and RNA for analysis of known and potential new factors that put healthy ageing at risk*. The study will collect and conduct a wide range haematological and biochemical tests and the bio-banked samples will provide a resource to investigate novel markers of ageing and disease processes. The collection will act as a resource for future genetic studies, since well-characterised (well-phenotyped) populations are of immense value for collaborative genetic population studies. The collection of DNA and RNA will also allow genetic and functional studies to examine pathological pathways in disease processes associated with ageing.

4. *Providing data for prioritised future health planning*. The collection of comprehensive population health data (including environmental and genetic risk factors) will contribute to the development of strategies for facilitating healthy and productive ageing in the general community.

5. *Helping to define healthy ageing.* Using a multi-dimensional clustering approach, the study will explore clusters of resilient individuals with low illness and high physical and cognitive functioning. It will provide important information about the factors associated with optimal ageing. Thus, it will meet the call for more refined outcome measures in the definition of healthy ageing [62] but also inform the understanding of the mechanisms and life-course of resilience and frailty or disability.

In contrast to a number of large population surveys that have been limited in the range of data that can be collected for each subject, the BHAS aims to characterize individuals using questionnaire data, physical examination, biomarkers and genetic markers with a view to longitudinal follow-up, including incident illness, change in quality of life and function and health care utilisation. Given the unique opportunity for extensive data linkage that exists in Western Australia, subject cross-sectional and longitudinal data will be related to hospital admissions and (in future studies) mortality.

The results from the BHAS will provide a better understanding of the ageing process and a basis for planning future health strategies for an ageing population. This will be achieved by exploration of how combinations of co-morbidities (and their risk factors) interact and impact on function. The BHAS will provide health information on the frequency with which co-morbidities cluster within individuals, which co-morbidities have the most effect on function and how some co-morbidities modify (either positively or negatively) the impact of others on function. Previous studies that have reported the effects of combinations of diseases have shown important modification effects of concurrent disease but have been retrospective, examined a limited range of diseases and have been less focused on specific age groups. The BHAS is specifically designed to prospectively assess an extensive range of co-existing morbidities and determine their effects on overall function in an older population sample.

The BHAS will establish a framework that will enable the development of interventions and health promotion programs that can be trialled, and their impact assessed quantitatively, in a general population sample of ageing adults.

Finally, the BHAS provides feed-back to each participant on clinically relevant outcomes (see Tables 1 and 2) with advice to take results to their usual medical practitioner. This includes blood and other test results all of which are scrutinised by a chief investigator. Where indicated participants are contacted directly to inform them of abnormal results requiring immediate attention.

### Limitations of the study

The BHAS has a focus on studying the cumulative impacts on function of linked or apparently disparate illnesses within individuals at baseline and during ageing in planned follow-up studies. Therefore the phenotyping within individuals is time-consuming and labour intensive and is confined to “baby boomers” at the onset of ageing. This moderately-sized cohort will provide good power cross-sectionally and longitudinally to study effects of common risk factors and illnesses but the study is not well-powered to examine associations for uncommon events or risk factors and minor effect modification.

The population sample is drawn from the Busselton Shire and is confined to a relatively narrow age-range, is racially very homogeneous (<1% race other than Western European) and all individuals currently live in a homogeneous climactic environment. Therefore generalisation to other populations, ages and racial groups may have limitations. However, pilot data from the first 300 subjects show prevalence values for common risk factors such as obesity and overweight to be almost identical to those in the recent nationally representative Australian Health Survey (4364.0.55.003 - Australian Health Survey: Updated Results, 2011–2012).

The Busselton Shire has been the scene of repeated cross-sectional health surveys since 1966 although there have been only 2 major surveys since 1990 in 1994–95 and 2005–08. As such a number of participants in the BHAS have participated in a previous survey or at least will be aware of previous survey activity. As stated above, in the current and also the previous major surveys, all participants are provided with results of clinically relevant tests and where necessary are contacted by study investigators with advice to attend their usual practitioner for appropriate follow-up. These factors may also affect generalizability, particularly with disease incidence and prevalence. An earlier analysis of the impact of the surveys on mortality and hospital morbidity found no conclusive impact of the surveys [63].

Whilst these limitations to the generalisability of the Busselton population to the whole Australia population exist, they are more of a concern for generalization of absolute risk and prevalence estimates and of less concern for generalizability of relationship effect estimates which are the primary focus of the study.

## Competing interests

The authors declare that they have no competing interests.

## Authors’ contributions

All authors have contributed substantially to the design of this protocol. AJ, MH and LS drafted the manuscript based on the BHAS Research Design and Plan written by all BHAS investigators. All authors read, revised and approved the final manuscript.

## Pre-publication history

The pre-publication history for this paper can be accessed here:

http://www.biomedcentral.com/1471-2458/13/936/prepub
